# IS-Pro-based pathogen detection in explanted heart valves of suspected endocarditis patients

**DOI:** 10.1128/spectrum.03984-25

**Published:** 2026-03-09

**Authors:** Patricia Bartsch, Oliver Krebs, Christa Augustin, Martin Aepfelbacher, Holger Rohde, Benjamin Berinson

**Affiliations:** 1 Institute for Medical Microbiology, Virology and Hygiene, University Medical Center Hamburg-Eppendorf 37734, Hamburg, Germany; 2 Institute of Legal Medicine, Center for Diagnostics, University Medical Center Hamburg-Eppendorf 37734, Hamburg, Germany; University of Mississippi Medical Center, Jackson, Mississippi, USA

**Keywords:** infectious endocarditis, pathogen detection, culture, IS-Pro, molecular assays, 16S rDNA PCR syndromic panel PCR, Biofire Film Array JI panel

## Abstract

Accurate pathogen identification in infective endocarditis (IE) is limited, particularly in culture-negative cases. This study evaluated the performance of the Molecular Culture ID (MC-ID) assay in heart valve specimens from patients with suspected IE. Fifty-seven valve specimens from patients with suspected IE were analyzed using conventional culture, 16S/18S rDNA sequencing (SepsiTest, Molzym, Germany), the Biofire Joint Infection Panel (BJP; bioMérieux, Marcy l'Étoile, France), and MC-ID, with previously reported results as comparators. Twelve specimens (21.1%) were culture-positive, and 45 (78.9%) were culture-negative. MC-ID showed 83.3% positive agreement and 44.4% negative agreement with culture, achieving species-level concordance in 8/12 cases. Three culture-positive/MC-ID-negative results reflected organisms outside the assay’s detection range or limited taxonomic resolution. MC-ID detected pathogens in 25/45 (55.5%) culture-negative specimens; 21 were corroborated by PCR and/or BJP. Discrepancies largely reflected polymicrobial infections or taxonomic resolution limits. MC-ID identified pathogens in four PCR-/BJP-negative specimens, of which three likely were of clinical significance. MC-ID was negative in two culture-negative, PCR-/BJP-positive specimens. MC-ID can improve pathogen detection in both culture-positive and culture-negative IE, complementing conventional and molecular diagnostics and providing a comprehensive microbiological assessment in suspected IE cases.

**IMPORTANCE:**

Infective endocarditis (IE) remains difficult to diagnose, especially when blood and tissue cultures fail to identify a pathogen. Accurate organism detection is essential for guiding targeted therapy and reducing reliance on broad-spectrum antibiotics. This study shows that the Molecular Culture ID (MC-ID) assay markedly improves diagnostic yield in culture-negative valve specimens, frequently identifying pathogens missed by conventional and molecular tests. MC-ID also clarifies cases complicated by polymicrobial infection or limited taxonomic resolution, providing species-level or clinically actionable identification in many instances. By complementing existing diagnostic methods and expanding detection beyond their constraints, MC-ID offers a more comprehensive microbiological assessment of suspected endocarditis. Its integration into diagnostic workflows has the potential to enhance clinical decision-making and improve management of this high-risk condition.

## INTRODUCTION

Diagnosing infective endocarditis (IE) remains challenging, particularly in patients exposed to anti-infective therapy or with the presence of fastidious, difficult-to-culture organisms (e.g., *Bartonella* spp.) (1, 2). Accurate identification of the causative pathogens, though, is essential for the targeted treatment of IE (3). Therefore, as a complement to blood cultures, culture-independent methods for pathogen detection have lately attracted much interest. Although currently of limited value for peripheral blood analysis, methods like 16S/18S rDNA PCR or syndromic panel assays have successfully been employed to identify pathogens from explanted, native, or prosthetic heart valves (4). Notably, in this context, the diagnostic sensitivity of molecular assays is superior as compared to that of culture (5), suggesting integration of molecular techniques into routine workflows, aligning with current Duke’s criteria (1) to diagnose IE. Indeed, the introduction of commercial, fully integrated PCR assays allowing for the detection of multiple pathogens in clinical specimens in parallel (i.e., syndromic panel PCRs [spPCR]) has opened the opportunity for wider implementation of molecular assays in routine workflows. However, spPCR assays only cover a restricted number of organisms. The associated risk of false-negative results in infectious syndromes caused by a wide variety of pathogens therefore favors the use of more unbiased approaches in which 16S rDNA is amplified by PCR and species are deduced from the sequencing of resulting amplicons (4, 6, 7). These assays, though, suffer from potential lower sensitivity and more complex workflows, including the need for sequencing of amplicons (5). Here, we evaluated the diagnostic accuracy of the recently developed Molecular Culture ID (MC-ID) (8–10) assay to detect and identify pathogens in explanted heart valves. MC-ID is a rapid, PCR-based microbial identification approach based on the IS-Pro technology, which combines the analysis of length polymorphisms in the 16S–23S rRNA intergenic spacer region with phylum-specific fluorescently labeled primers to detect and identify bacteria at the species level in a culture-independent manner (10). The assay has the ability to identify a broad range of species, but it also has known limitations since some classes or phyla (e.g., Mollicutes, Chlamydiaceae, and Spirochaetes) are not detected. Results of the MC-ID assay were compared to those of conventional culture, 16S/18S rDNA PCR (UMD-SelectNA), and spPCR (Biofire JI panel; BJP).

## MATERIALS AND METHODS

### Tissue specimens, standard-of-care diagnostics, and molecular reference testing

This study was conducted on 57 heart valve tissue specimens collected from patients with suspected infective endocarditis. All specimens underwent standard-of-care (SOC) diagnostics and were additionally analyzed using 16S/18S rDNA PCR and subsequent sequencing of resulting amplicons (SepsiTest-UMD, Molzym, Bremen, Germany, referred to as 16S/18S rDNA assay) and the Biofire Filmarray Joint Infection Panel (bioMérieux, Marcy l'Étoile, France, referred to as BJP). The diagnostic procedures and corresponding results have been reported previously (5). In brief, tissue homogenization was performed using the IKA Ultra Turrax Tube Drive control homogenizer (IKA Werke GmbH & Co. KG, Staufen, Germany). After bead beating, 50–100 µL of the tissue homogenate was plated onto Columbia sheep blood agar and chocolate agar, as well as on Schaedler anaerobic agar (all from Oxoid, Basingstoke, UK). All aerobic incubation procedures were carried out at 37°C and 5% CO_2_ for up to 14 days, and anaerobic incubation was performed for up to 7 days. Plates were evaluated for growth after 24 h, 48 h, 7 days, and 14 days. All pathogens grown by culture were subjected to species identification using a matrix-assisted laser desorption/ionization time of flight (MALDI-ToF) instrument (Microflex, Bruker Daltonics, Bremen, Germany). 16S/18S rDNA analysis was performed according to a recent publication (11). In brief, 1 mL of homogenized specimen was depleted of human DNA, and subsequently bacterial DNA was isolated according to the manufacturer’s protocol. A PCR for amplification of 16S/18S rDNA was run on a LightCycler 480 instrument. PCR amplicons were purified (QIAquick PCR purification kit; Qiagen, Hilden, Germany) and sent for Sanger sequencing. 16S/18S sequences were analyzed with the web-based SepsiTest-BLAST application (Molzym). The BJP is approved for the analysis of synovial fluid. BJP analysis was performed essentially according to the manufacturer’s instructions, with the exception that tissue homogenate was used instead of synovial fluid. In brief, the manufacturer-provided hydration solution was loaded to the pouch, and 200 µL of the heart valve homogenate was mixed with the provided sample buffer. This mixture was loaded to the pouch, which then was inserted into the instrument, and the assay was run by the system. MC-ID analysis was performed on the same specimens as BJP and the 16S/18S rDNA assay; however, independent DNA extractions were performed from left-over tissue homogenates that had been stored at −80°C. For consistency and ease of cross-reference, this present report applies the same specimen designation as used in that publication (5).

### DNA extraction and MC-ID analysis

One hundred microliters of the left-over, bead-beaten homogenized heart valve material (5) was added to Bacterial Shock Buffer 1 (inBiome, Amsterdam, The Netherlands) and incubated for 10 min at 800 rpm and 95°C. After addition of 25 µL of Bacterial Shock Buffer 2 (inBiome, Amsterdam, The Netherlands) per sample, DNA was eluted on a MagNa Pure 96 instrument using the MagNa Pure DNA and Viral NA Small Volume Kit (Roche, Basel, Switzerland) and the Pathogen Universal 200 extraction program to give a final volume of 50 µL. Further processing of the sample was done according to the MC-ID Instructions for Use (inBiome, Amsterdam, The Netherlands). Amplification was carried out on a ProFlex PCR-System instrument (Thermo Fisher Scientific). Amplicons were separated on a SeqStudio Genetic Analyzer (Thermo Fisher Scientific). MC-ID results were generated using the Antoni software platform (inBiome, Amsterdam, The Netherlands).

### Amplicon sequencing

In the cases in which the standard MC-ID workflow was unable to resolve a species, MC-ID PCR amplicons were sequenced on a MinION device (Oxford Nanopore Technologies). PCR products were diluted 1,000-fold and re-amplified using the 2 MC-ID primer sets. Barcoded libraries were prepared (SQK-LSK109, EXP-NBD196 kits) and sequenced on R9 flow cells. Reads containing MC-ID forward and reverse primers were extracted from the FASTA files and used to generate consensus sequences. These sequences were classified via BLAST (NCBI nr/nt and whole-genome sequencing [WGS] databases), retaining hits with >95% query coverage and identity.

## RESULTS

A total of 57 heart valve specimens from 57 individual patients with suspected IE were included in the study. All specimens were analyzed using conventional culture, the 16S/18S rDNA assay, and the BJP, and results have been described previously (5) (Table S1).

Among the 57 specimens analyzed, 12 (21.1%) were culture-positive, while the remaining 45 (78.9%) were culture-negative. The overall positive percent agreement (PPA) of MC-ID, defined as a positive result in culture-positive samples, was 83.3% (10/12), while the negative percent agreement (NPA), defined as concordance with culture-negative samples, was 44.4% (20/45). Of the 12 culture-positive samples, MC-ID achieved species-level concordance with culture in eight cases (66.6%, Table S1). In one of four samples (sample 14), MC-ID called the *Streptococcus pneumoniae/mitis* group, while culture grew the *Streptococcus sanguinis* group. In a second sample (sample 86), MC-ID was only able to differentiate culture-proven *Streptococcus equi* to the phylum level (Firmicutes, Actinobacteria, Fusobacteria, and Verrucomicrobia bacteria [FAFV], Table 1). Two remaining MC-ID negative, culture-positive cases (sample 6 and 54) can reasonably be explained by limitations of the assay as the organisms detected by culture (*Mycoplasma hominis* [sample 6] and *Candida albicans* [sample 54]) fall outside the MC-ID detection ability (Table 1).

**TABLE 1:** Comparison of MC-ID results with reference methods: discrepant results

Study number	Culture	16S/18S rDNA PCR	spPCR (Biofire Joint infection panel; BJP)	IS-pro (Molecular Culture-ID)
Culture-positive samples; discrepant or negative MC-ID results				
6	*M. hominis*	*M. hominis*	Negative	Negative
14	*S. sanguinis* group	*S. gordonii*	*Streptococcus* spp.	*S. pneumoniae/mitis* group
54	*C. albicans*	*C. albicans*	*C. albicans*	Negative
86	*S. equi*	*S. equi*	*Streptococcus* spp.	Unknown FAFV bacteria^*a*^
Culture-negative cases; discrepant results between MC-ID and 16S rDNA PCR and/or BJP				
20	Negative	*Corynebacterium* species *(pseudokroppenstedtii/kroppenstedtii*)	*E. coli*	*E. coli/Shigella* spp. and *C. striatum*
31^*b*^	Negative	*S. mitis* group	*Streptococcus* spp.	*S. epidermidis/S. sanguinis*
65^*c*^	Negative	*L. gervieae/formosensis*	Negative	*R. dentocariosa*
76	Negative	*E. faecalis*	*E. faecalis*	*E. faecalis* and *S. bovis group/S. intermedius*
88	Negative	*S. sanguinis* group	*Streptococcus* spp.	*S. pneumoniae/mitis* group
Culture-, 16S rDNA-, and BJP-negative cases; MC-ID-positive				
18^*d*^	Negative	Negative	Negative	*S. pneumoniae/mitis* group and unknown FAFV bacteria^*a*^^,*e*^
32	Negative	Negative	Negative	*E. coli/Shigella* spp. and unknown Bacteroidetes bacteria^*f*^
68^*d*^^,*g*^	Negative	Negative	Negative	*S. pneumoniae/mitis* group
83^*g*^	Negative	Negative	Negative	*S. sanguinis*
Culture- and MC-ID-negative cases; 16S rDNA- and/or BJP-positive				
28	Negative	Negative	*E. faecalis*	Negative
63	Negative	*S. aureus*	*S. aureus*	Negative

^*a*^ FAFV is the abbreviation for Firmicutes, Actinobacteria, Fusobacteria and Verrucomicrobia bacteria.

^*b*^ The software’s capacity to analyze the data was impeded by the medium–high abundance of bacteria, rendering it unable to differentiate between *S. sanguinis* and *S. epidermidis*. However, a manual analysis revealed the presence of Streptococci in the sample.

^*c*^ *Lactococcus garvieae/formosensis* was regarded as a true positive result as consistent species was recovered from BC taken from the respective patient. The species is a BJP off-panel pathogen, explaining the negative result. *Rothia dentocariosa* is until now not distinguishable from *L. garvieae/formosensis* by MC-ID.

^*d*^ Evidence for infective endocarditis on the basis of floating material in sonography.

^*e*^ Unknown FAFV bacteria were identified as *Lactobacillus (para)gasseri*, *Paracoccus marcusii,* and *Limosilactobacillus vaginalis* by amplicon sequencing.

^*f*^ Unknown Bacteriodetes bacteria were identified as *Bacteroides vulgatus* by amplicon sequencing.

^*g*^ Strong evidence for infective endocarditis on the basis of positive blood cultures.

The ability to identify pathogens in culture-negative specimens was of particular interest in this study. MC-ID identified pathogens in 55.5% (25/45) of all culture-negative cases. In 16/25 cases, MC-ID results were fully corroborated by findings from 16S/18S rDNA assay and/or BJP analysis (Table S1). In five samples, MC-ID assay results did not fully align with findings from the 16S/18S rDNA assay and/or BJP (samples 20, 31, 65, 76, and 88; Table 1). In two of these samples (sample 20 and 76), discrepancies were related to the detection of polymicrobial infections not fully covered by the 16S/18S rDNA assay and/or BJP. In case 20, for example, the 16S/18S rDNA assay identified *Corynebacterium* spp., whereas BJP detected *E. coli*, which clinicians interpreted as the causative pathogen based on available blood culture results. Notably, MC-ID identified both organisms, thereby providing a more comprehensive characterization of the sample (Table 1). In sample 65, MC-ID identified *Rothia dentocariosa*, which by MC-ID currently cannot be distinguished from *Lactococcus gervieae/formosensis*, as identified by the 16S/18S rDNA assay. In sample 88, MC-ID identified the *S. pneumoniae/mitis* group, whereas the 16S/18S rDNA assay and BJP identified *S. sanguinis* and *Streptococcus* spp., respectively.

In four samples (samples 18, 32, 68, and 83) that were negative by both the 16S/18S rDNA assay and BJP analysis, MC-ID yielded positive results (Table 1); however, the clinical significance of these findings remains uncertain and needs to be cross-validated by findings from independent specimens (e.g., blood cultures).

MC-ID failed to detect a pathogen in two culture-negative specimens that were positive by the 16S/18S rDNA assay and/or BJP. Pathogens identified in these samples (*S. aureus* and *E. faecalis*, respectively) were deemed true positives by the clinicians (Table 1).

## DISCUSSION

Identifying the causative pathogen is essential for optimal therapy in IE, yet blood and valve culture-negative cases remain a significant challenge (1, 2). Culture-independent diagnostics offer a promising solution, and availability of assays adhering to IVDR (*in vitro* diagnostic medical device regulation) standards now allows broader molecular testing outside specialized laboratories. In this study, we evaluated the analytical performance of the MC-ID assay for pathogen detection in explanted heart valves from suspected cases of infective endocarditis and compared results with those of standard culture, a commercial 16S/18S rDNA assay, and the syndromic PCR assay BJP.

The main strength of molecular assays is their ability to detect pathogens in culture-negative cases, including patients pretreated with antibiotics or infections caused by fastidious organisms (12, 13). Consistent with findings from other culture-independent diagnostic methods (4, 8, 14), the MC-ID assay demonstrated markedly higher sensitivity than culture, identifying pathogens in 25/45 culture-negative samples. In 21 of these 25 samples, MC-ID detected the same species as the 16S/18S rDNA assay and BJP, highlighting its analytical reliability. The robustness of the MC-ID assay is underscored by a recent study in which the diagnostic accuracy of the assay was tested in 591 synovial fluid samples (9). In that study, the MC-ID assay demonstrated a PPA of 90.6% and an NPA of 87.7%, with 80% concordance at the species level. Notably, the assay detected pathogens in 83 culture-negative specimens, underscoring its potential to substantially increase the proportion of infections with an established etiologic diagnosis (9). Indeed, in our study, the MC-ID assay identified pathogens in 4 samples that were negative by all culture and molecular reference assays. In 2 of these (samples 68 and 83), pathogen identifications were corroborated by concordant blood culture results. In sample 68, MC-ID identified the *S. pneumoniae/mitis* group, which aligns with blood cultures that grew *Streptococcus gordonii*. Similarly, in case 83, MC-ID called *S. sanguinis*, which is in concordance with isolates identified from blood cultures. In 1 case (sample 18), MC-ID detected viridans streptococci, at least representing a plausible pathogen in the context of infective endocarditis. In sample 32, MC-ID detected *E. coli/Shigella* spp. along with additional FAFV, yet a chart review revealed no clinical suspicion of endocarditis. Clearly, clinical relevance of pathogens identified by MC-ID that were not verified by alternative methods—including other FAFV bacteria—remains uncertain.

While on one hand these results demonstrate the potential additional value of MC-ID analysis in IE diagnostics, it is important to stress that on the other hand, MC-ID analysis failed to identify clinically significant pathogens in four samples that were identified by either culture and/or reference molecular assays. Thus, no currently available commercial assay clearly outperforms the others in diagnostic accuracy. Instead, each test exhibits distinct, assay-intrinsic limitations that must be taken into account. For example, the MC-ID assay cannot identify or distinguish Mollicutes (e.g., *Mycoplasma* species), Chlamydiae, and Spirochaetes; the BJP is constrained by its limited organism panel (5); and the 16S/18S rDNA assay may be affected by reduced sensitivity (5, 15). Of note, a general drawback of all culture-independent methods evident from our analysis relates to specific limitations in differentiation of viridans streptococci due to their close genetic relatedness with high sequence conservation within the 16S rDNA gene region, highlighting the importance of combining phenotypic, genotypic, and clinical data when interpreting results, particularly for taxonomically challenging bacterial groups (16).

By integrating broad pathogen coverage with rapid turnaround time (~5 h), the MC-ID assay has potential advantages over 16S/18S rDNA assays or spPCR, that is, 16S/18S rDNA assays offer broad pathogen coverage, but typically require >24 h time-to-result (17), while spPCR assays offer potential faster turnaround times (e.g., ~ 60 min for the BJP assay), but are inherently restricted by a narrower target spectrum, potentially delaying diagnosis in infections caused by rarer pathogens. It should be stressed that the BJP assay is not approved for the use of heart valve tissue homogenates, and its clinical use would require a full validation. In general, the clinical utility of the MC-ID and other molecular assays must be contextualized within diagnostic and antimicrobial stewardship frameworks: test requests should be guided by predefined criteria, and results interpreted in conjunction with clinical findings to avoid misdiagnosis or inappropriate therapy. Importantly, while the MC-ID assay is compatible with commonly available thermocyclers, the requirement for high-resolution amplicon separation on a capillary electrophoresis instrument may limit adoption in some laboratories, highlighting a practical constraint on implementation.

This study has some limitations. Importantly, the MIC-ID analysis was performed on the same stored tissue specimen homogenates as the molecular reference assays. However, new DNA extractions were necessary as there were no leftovers after 16S/18S rDNA assay and BJP analysis. This may explain some of the discrepancies between the MIC-ID assay results and those of reference molecular assays. In addition, the study is limited by the monocentric study design, the small number of samples, and the limited range of species included.

Taken together, this study supports the idea that molecular assays play an important role in pathogen detection in IE. Further prospective studies are warranted that comparatively analyze the analytical performance of available commercial molecular assay for pathogen detection directly from heart valve tissues. Moreover, such studies need to assess the clinical impact of any molecular assays for pathogen detection from heart valves on antimicrobial decision-making and outcomes as well as health-economic effects.

## SUPPLEMENTAL MATERIAL

Table S1 spectrum.03984-25-s0001.docx Per-specimen results of culture, 16S/18S rDNA PCR, BJP, and MC-ID.

ASM does not own the copyrights to Supplemental Material that may be linked to, or accessed through, an article. The authors have granted ASM a non-exclusive, world-wide license to publish the Supplemental Material files. Please contact the corresponding author directly for reuse.
