# Supplementary material for: IS-Pro-based pathogen detection in explanted heart valves of suspected endocarditis patients
Source: Microbiol Spectr. 2026 Mar 9;14(4):e03984-25. doi: 10.1128/spectrum.03984-25 (PMC13055294; doi:10.1128/spectrum.03984-25)
Supplement: Table S1 — Per-specimen results of culture, 16S/18S rDNA PCR, BJP, and MC-ID. [file spectrum.03984-25-s0001.docx]

**Supplementary Table S1: Per specimen results of culture, 16S/18S rDNA PCR, BJP and MC-ID**

| **Study number** | **Culture** | **16S/18S rDNA PCR** | **BJP** | **MC-ID** |
| --- | --- | --- | --- | --- |
| 3 | Negative | *S. mutans* | *Streptococcus spp.* | *S. mutans* |
| 5 | Negative | Negative | Negative | Negative |
| 6 | *M. hominis* | *M. hominis* | Negative | Negative |
| 7 | Negative | Negative | *E. faecalis* | *E. faecalis* |
| 9 | Negative | Negative | Negative | Negative |
| 13 | Negative | Negative | Negative | Negative |
| 14 | *S. sanguinis* Group | *S. gordonii* | *Streptococcus spp.* | *S. pneumoniae/mitis* Group |
| 17 | Negative | *S. dysgalactiae* | *Streptococcus spp.* | *S. dysgalactiae* |
| 18^a^ | Negative | Negative | Negative | *S. pneumoniae/mitis* Group and unknown FAFV bacteria^b, c^ |
| 19 | Negative | *S. mitis* Group | Negative | *S. pneumoniae/mitis Group* |
| 20 | Negative | *Corynebacterium species (kroppenstedtii/pseudokroppenstedtii)* | *E. coli* | *E.coli /Shigella spp.* and *C. striatum* |
| 21 | Negative | Negative | Negative | Negative |
| 22 | Negative | Negative | Negative | Negative |
| 23 | Negative | *E. faecalis* | *E. faecalis* | *E. faecalis* |
| 24 | *S. epidermidis* | *S. epidermidis/caprae/capitis* | Negative | *S. epidermidis* |
| 25 | *E. faecalis* | *E. faecalis* | *E. faecalis* | *E. faecalis* |
| 28 | Negative | Negative | *E. faecalis* | Negative |
| 31^d^ | Negative | *S. mitis* Group | *Streptococcus spp.* | *S. epidermidis/ S.sanguinis* |
| 32^e^ | Negative | Negative | Negative | *E. coli/ Shigella spp.* and unknown Bacteroidetes bacteria |
| 34 | Negative | *S. agalactiae* | *S. agalactiae* | *S. agalactiae* |
| 37 | *S. aureus* | *S. aureus* Complex | *S. aureus* | *S. aureus* |
| 39 | *S.equinus/bovis* Group | *S. equinus/bovis* Group | *Streptococcus spp.* | *S. bovis* Group */ S. intermedius* |
| 41 | Negative | Negative | Negative | Negative |
| 42 | Negative | *S. aureus* Complex | *S. aureus* | *S. aureus* |
| 43 | *S. aureus* | *S. aureus* Complex | *S. aureus* | *S. aureus* |
| 46 | Negative | *S. anginosus* Group | *Streptococcus spp.* | *S. intermedius/anginosus* |
| 47 | Negative | Negative | Negative | Negative |
| 48 | Negative | Negative | Negative | Negative |
| 50 | Negative | Negative | Negative | Negative |
| 51 | *S. aureus* | *S. aureus* Complex | *S. aureus* | *S. aureus* |
| 52 | Negative | Negative | Negative | Negative |
| 54 | *C. albicans* | *C. albicans* | *C. albicans* | Negative |
| 55 | Negative | Negative | *E. coli* | Negative |
| 56 | Negative | *S. aureus* Complex | *S. aureus* | *S. aureus* |
| 57 | Negative | *S. mitis* Group | *Streptococcus spp.* | *S. pneumoniae/mitis* Group |
| 60 | Negative | *S. mitis* Group | *Streptococcus spp.* | *S. pneumoniae/mitis* Group |
| 61 | Negative | Negative | Negative | Negative |
| 63 | Negative | *S. aureus* Complex | *S. aureus* | Negative |
| 64 | Negative | *S. sanguinis* Group | *Streptococcus spp.* | *Streptococcus sanguinis* |
| 65^f^ | Negative | *L. garvieae/formosensis* | Negative | *R. dentocariosa* |
| 67 | Negative | *S. pneumoniae* | *S. pneumoniae* | *S. pneumoniae/mitis* Group |
| 68 ^a, g^ | Negative | Negative | Negative | *S. pneumoniae/mitis* Group |
| 69 | Negative | Negative | Negative | Negative |
| 70 | Negative | Negative | Negative | Negative |
| 71 | Negative | *S. aureus* Complex | *S. aureus* | *S. aureus* |
| 75 | *S. lugdunensis* | *S. lugdunensis* | *S. lugdunensis* | *S. lugdunensis* |
| 76 | Negative | *E. faecalis* | *E. faecalis* | *E. faecalis* and *S. bovis* Group*/ S. intermedius* |
| 83^g^ | Negative | Negative | Negative | *S. sanguinis* |
| 84 | Negative | Negative | Negative | *Negative* |
| 85 | Negative | Negative | Negative | Negative |
| 86 | *S. equi* | *S. equi* | *Streptococcus spp.* | Unknown FAFV bacteria |
| 87 | Negative | Negative | Negative | Negative |
| 88 | Negative | *S. sanguinis* Group | *Streptococcus spp.* | *S. pneumoniae/mitis* Group |
| 89 | Negative | Negative | Negative | Negative |
| 90 | Negative | *S. agalactiae* | *S. agalactiae* | *S. agalactiae* |
| 91 | *S. aureus* | *S. aureus* Complex | *S. aureus* | *S. aureus* |
| 92 | Negative | *C. striatum* | Negative | *C. striatum* |

^a^ Evidence for infective endocarditis on the basis of floating material in sonography.

^b^ FAFV is the abbreviation for Firmicutes, Actinobacteria, Fusobacteria and Verrucomicrobia bacteria.

^c^ Unknown FAFV bacteria were identified as *Lactobacillus (para) gasseri*, *Paracoccus marcusii* and *Limosilactobacillus vaginalis* by amplicon sequencing.

^d^ The software's capacity to analyze the data was impeded by the medium-high abundance of bacteria, rendering it unable to differentiate between *S. sanguinis* and *S. epidermidis*. However, a manual analysis revealed the presence of Streptococci in the sample.

^e^ Unknown bacteriodetes bacteria was identified as *Bacteroides vulgatus* by amplicon sequencing.

^f^ *Lactococcus garvieae/formosensis* is a BJP off-panel pathogen, explaining the Negative result. *Rothia dentocariosa* is until now not distinguishable from *L. garvieae/formosensis* by MC-ID method.

^g^ Strong evidence for infective endocarditis on the basis of positive blood cultures.
